# Scale Dependence of Female Ungulate Reproductive Success in Relation to Nutritional Condition, Resource Selection and Multi-Predator Avoidance

**DOI:** 10.1371/journal.pone.0140433

**Published:** 2015-10-16

**Authors:** Jared F. Duquette, Jerrold L. Belant, Nathan J. Svoboda, Dean E. Beyer, Patrick E. Lederle

**Affiliations:** 1 Carnivore Ecology Laboratory, Forest and Wildlife Research Center, Mississippi State University, Mississippi State, Mississippi, United States of America; 2 Michigan Department of Natural Resources, Wildlife Division, Marquette, Michigan, United States of America; 3 Michigan Department of Natural Resources, Wildlife Division, Lansing, Michigan, United States of America; University of Sassari, ITALY

## Abstract

Female ungulate reproductive success is dependent on the survival of their young, and affected by maternal resource selection, predator avoidance, and nutritional condition. However, potential hierarchical effects of these factors on reproductive success are largely unknown, especially in multi-predator landscapes. We expanded on previous research of neonatal white-tailed deer (*Odocoileus virginianus*) daily survival within home ranges to assess if resource use, integrated risk of 4 mammalian predators, maternal nutrition, winter severity, hiding cover, or interactions among these variables best explained landscape scale variation in daily or seasonal survival during the post-partum period. We hypothesized that reproductive success would be limited greater by predation risk at coarser spatiotemporal scales, but habitat use at finer scales. An additive model of daily non-ideal resource use and maternal nutrition explained the most (69%) variation in survival; though 65% of this variation was related to maternal nutrition. Strong support of maternal nutrition across spatiotemporal scales did not fully support our hypothesis, but suggested reproductive success was related to dam behaviors directed at increasing nutritional condition. These behaviors were especially important following severe winters, when dams produced smaller fawns with less probability of survival. To increase nutritional condition and decrease wolf (*Canis lupus*) predation risk, dams appeared to place fawns in isolated deciduous forest patches near roads. However, this resource selection represented non-ideal resources for fawns, which had greater predation risk that led to additive mortalities beyond those related to resources alone. Although the reproductive strategy of dams resulted in greater predation of fawns from alternative predators, it likely improved the life-long reproductive success of dams, as many were late-aged (>10 years old) and could have produced multiple litters of fawns. Our study emphasizes understanding the scale-dependent hierarchy of factors limiting reproductive success is essential to providing reliable knowledge for ungulate management.

## Introduction

Behaviors of prey living in seasonal environments are predicted to reflect the spatial and temporal scales at which factors limiting their survival occur [1, 2]. Prey are predicted to avoid factors limiting their survival at larger scales [3] because a limiting factor (e.g., predation) should continue to dominate prey behavior at successively finer scales until another limiting factor (e.g., food) becomes more influential to survival [4]. However, the prediction of broad-scale limitation is not always empirically supported because spatiotemporal heterogeneity of several factors can cumulatively limit survival at different spatial and temporal scales [2]. For example, when food distribution is more spatially heterogeneous across the landscape than within home ranges, but predation risk is more spatially heterogeneous within home ranges than across the landscape, prey survival should be influenced by food at the landscape scale and predator avoidance at the home range scale [2]. Therefore, assessing if survival is limited at only one spatial or temporal scale could be inadequate to observe if multiple factors cumulatively limit survival at multiple scales.

To understand how prey increase their reproductive success, studies often assess resource use, predation risk, or their interaction among multiple spatial or temporal scales [5–8]. While prey behaviors may not be necessarily related to these predetermined scales, multi-scale analyses allow us to interpret biological processes which can be useful information for conservation and management of the species [9]. For example, survival may be limited by vegetation characteristics at the home range scale [10], but predation at the landscape scale [3], which require different management considerations. Understanding the hierarchal influence of limiting factors on prey reproductive success should therefore include investigating how prey allocate their resource selection and predator avoidance behaviors across a landscape [11, 12] and at progressively finer spatial scales (e.g., home range; [13]).

Spatiotemporal variation in predation risk can limit the space use and nutritional condition of ungulates based on their ‘fear’ of predators across the landscape [11, 13, 14]. Therefore, behavioral trade-offs between resources and predation risk are especially important to ungulates post-parturition when neonates are most susceptible to predation, which can affect population growth [15, 16]. Consequently, we would expect parturient female ungulates to avoid resources (e.g., grassland) with greater predation risk across a landscape to maximize the likelihood of neonate survival (i.e., reproductive success; [17, 18]). However, the immense nutritional strain neonates place on females [16, 19, 20] can cause females to select vegetation that provides greater nutritional gain, rather than select resources where neonates are more likely to survive [21]. Severe weather, especially winter [16], can further exacerbate the extent females can nutritionally support neonates [22, 23] and the increased phenology of vegetation needed by neonates for hiding cover from predators [11, 14]. To balance nutritional demands with reducing predator detection of neonates [13, 14], female ungulates make behavioral trade-offs in resource selection and predator avoidance [24, 25] during the post-partum period.

To avoid predators, female ungulates rely on their knowledge or cues to variation in predation risk within resources across a landscape [26, 27], but this can be especially difficult in landscapes with multiple predator species. While each predator species may be more of a direct or indirect mortality risk [28, 29], females are forced to constantly assess the risk level each predator species presents within different areas (e.g., foraging and bedding; [30, 31]). While females may avoid areas with greater predation risk of predators presenting a direct mortality risk, alternative predators can capitalize on this behavior by increasing resource overlap with females, particularly when neonates present an energetically profitable food sources [13]. Therefore, assessing the resource selection and predator species-specific risk associated with resources can provide a valuable understanding of how parturient females maximize their reproductive success in a landscape with multiple predator species.

White-tailed deer (*Odocoileus virginianus*) abundance in the western Upper Peninsula of Michigan declined about 40% following 2 consecutive severe winters in the mid-1990s and has not recovered (Michigan Department of Natural Resources, unpublished data). Factors influencing the population decline are unknown. Deer pregnancy rates estimated from vehicle collisions during the 1990s were 80–95% [32] suggesting reproduction has not limited population growth in this region. Also, the number of antlerless deer observed by hunters during the 15-day firearm season has not decreased since 1994 [33]. However, predator abundances, particularly gray wolves (*Canis lupus*), have increased in this region [34] which could limit deer population growth [35, 36].

We expanded upon our previous home-range scale research [13] to assess if variation in daily or seasonal neonatal white-tailed deer (*Odocoileus virginianus*) survival was best explained by resource use, integrated risk of 4 mammalian predators, maternal nutritional effects, winter weather, hiding cover, or interactions among these variables during the post-partum period (14 May-31 Aug) across the landscape. This study expands our home range analyses [13] to assess what factors potentially influenced fawn survival at a seasonal and daily scale. These combined analyses allow us to compare how fawn survival was influenced at different spatial and temporal scales. Our study focused on survival of neonatal white-tailed deer because this age class was most influential to population growth [37] and fawn mortality is typically greatest during the first 3 months of life [15, 16]. We assumed fawn resource use and predation risk would reflect dam trade-offs in resource selection and predation risk avoidance [38]. We hypothesized that fawn survival would most benefit from dams avoiding predators at the landscape and seasonal scales to decrease overall likelihood that predators would encounter fawns and vigilance of dams toward predators [1, 17, 18]. However, we hypothesized that habitat use would be greater at the home range and daily scales because fawns require adequate nutrition from dams and sufficient hiding cover for survival. We developed 8 predictions describing resource use, multi-predator risk, and nutritional relationships to daily or cumulative seasonal effects on landscape-scale fawn survival [2, 13] under the null prediction that fawn survival was not influenced by any biological or environmental covariates (Table 1). These predictions include 2 predictions related to maternal nutritionally-mediated predation risk or ideal resource selection, in addition to the 6 described previously [13].

**Table 1:** Predictions used to assess daily or seasonal survival of neonate white-tailed deer (≤ 14 weeks of age) relative to resource use, predation risk, birth body mass, winter severity, and vegetation hiding cover at the landscape scale in the southcentral Upper Peninsula of Michigan, USA, 2009–2011.

Hypothesis	Prediction	Citations
Null	No biological or environmental factors were related to the mortality hazard	
Ideal resource use	A decrease in ideal resource use would increase the mortality hazard, irrespective of variation in predation risk	[84], [85]
Nutrition-mediated resource use	A decrease in the direct relationship between birth body mass and ideal resource use would have an increase in the mortality hazard.	[20], [22]
Predation risk	An increase in predation would increase the mortality hazard, irrespective of variation in resource use.	[3]
Maternal effects	Influence annual variation in survival through birth mass and winter weather severity or their interaction irrespective of other variables.	[16], [20], [22]
Hiding cover	Influences annual variation in survival through spring vegetation phenology, irrespective of other variables.	[27]
Weather-mediated predation risk	Winter severity and predation risk would have a direct relationship with an increase in the mortality hazard.	[11], [14]
Nutrition-mediated predation risk	Birth body mass and predation risk would have an inverse relationship with an increase in the mortality hazard.	[11], [22]
Non-ideal resource use	A decrease in ideal resource use which increases the mortality hazard with additive predation risk in those resources, further increasing the mortality hazard. Also, dam interpretation of habitat quality and their resource selection is not mediated by variation in predation risk.	[13], [86]
Ecological trap	Assumed similar resource use and predation risk relationships as “non-ideal resource use”, but assumed that resource use is mediated by the variation in predation risk perceived by dams leading to preference for poor-quality sink habitats.	[87], [88]
Weather-mediated ecological trap and nutrition	Assumed similar resource use and predation risk relationships as “Ecological trap”, but assumed that predation risk and maternal nutrition is mediated by the variation in winter weather experienced by dams leading to preference for poor-quality sink habitats.	[11], [22], [87], [88]

## Materials and Methods

### Ethics statement

Ethics of all capture and handling procedures were approved by the Mississippi State University Institutional Animal Care and Use Committee (#09–004) and animal capture and handling procedures followed guidelines established by the American Veterinary Medical Association and the American Society of Mammalogists [39]. Field studies did not involve endangered or protected species. Several private land parcels were used with landowner permission for field activities, but most were conducted on land owned by the Michigan Department of Natural Resources that granted access for our study. Data used in analyses can be obtained from the Supporting information files.

### Study area

Our study was conducted in the south-central Upper Peninsula of Michigan (45°43’47” N, 87°4’48” W; S1 Fig), which is topographically flat and has a mean elevation of 185 m above sea level. Lowland forest was the predominant land cover type and primarily consisted of eastern white cedar (*Thuja occidentalis*), eastern hemlock (*Tsuga canadensis*), and balsam fir (*Abies balsamea*) with areas of alder shrubs (*Alnus spp*.), but generally absent near roads. Remaining forest was upland or mixed with pine (*Pinus spp*.), aspen (*Populus spp*.), maple (*Acer spp*.), and birch (*Betula spp*.) trees. Grasses and shrubs were typically mixed and uncommon in the area. Cropland (mainly corn [*Zea spp*.] and soybeans [*Glycine spp*.]) and pasture accounted for about 13% of total landcover and were predominantly interspersed throughout the western half of the study area. Developed land was low density (0.09 km/km^2^) residential and recreational properties. Road density was 1.68 km/km^2^ and roads were predominantly paved, but several were gravel or soil. Permanent water (e.g., rivers and lakeshore) density was 1.17 km/km^2^. Mean monthly temperature from 2009 through 2011 ranged from 10.4°C in May to 19.0°C in August using a site-specific weather station sensor (model 107-L, Campbell Scientific Inc., Utah, USA). Remote camera surveys estimated annual adult and fawn deer density was 3.7–3.9/km^2^ and 0.6–1.3/km^2^, respectively [37]. Hair snare surveys estimated black bear (*Ursus americanus*) density was 0.14–0.19/km^2^ (Belant, J.L., unpublished data) and bobcat (*Lynx rufus*) density was 0.03/km^2^ [40]. Howl elicitation surveys estimated coyote (*Canis latrans*) density was 0.32–0.37/km^2^ [41] and winter track surveys of radiocollared wolves estimated wolf density was 0.012/ km^2^ (Petroelje, T.R. unpublished data).

### Fawn capture and monitoring

From May to July 2009–2011, we captured 129 neonatal fawns (estimated ≤ 15 days old; 69 males, 58 females, 2 unknown) opportunistically (*n* = 100) or with vaginal implant transmitter searches (*n* = 29; [42]) of radiocollared adult females throughout the study area. We used a spring scale to weigh fawns to the nearest 0.01 kg and then fit each with an expandable radiocollar. We identified sex, attached 2 ear tags, measured new hoof growth to estimate birth date and age [16], then released fawns at sites of capture. We estimated birth body mass of each fawn by subtracting the mean daily mass gain for northern, neonate white-tailed fawns (0.2 kg) from the capture mass [16]. We assumed capture and handling procedures and radiocollars analogous to [16] did not influence mortality risk of fawns.

We relocated fawns on a diel schedule up to 5 times/week from birth to 31 Aug each year using a truck-mounted 3 or 4 element Yagi antenna or aerial radiotelemetry using a 2 element antenna. We recorded 76% of relocations during diurnal hours (07:00–18:59) and 24% were recorded during nocturnal hours (19:00–06:59). We used Location of a Signal 4.0 software (Ecological Software Solutions LLC, Hegymagas, Hungary) to estimate fawn locations from the ground using ≥ 3 bearings recorded within 20 min [43]. We aerially estimated fawn locations by tightly circling over each individual radio signal ≥ 2 times at low altitude (i.e., ≤ 244 m) within 10 min and recording the location where we heard the loudest signal. We estimated ground-based telemetry error for personnel by placing randomly 5 radiocollars in forested or non-forested (e.g., pasture) vegetation and calculated mean ellipse error (2115 m^2^) from the known location of radiocollars and discarded recorded locations with error ellipses greater than the mean error.

When we detected radiocollars in mortality mode, we investigated sites within 8 hr and assessed if the mortality signal was due to fawn mortality or other causes (e.g., slipped radiocollar). We searched suspected mortality sites generally within 200 m of the radiocollar and expanded searches if we found evidence of mortality within this search zone. We recorded predator species-specific mortalities based on predation characteristics, carcass wounds, and site habitat characteristics, which we compared to published characteristics [44–47].

### Resource use

We used second-order selection analyses [48] to estimate resource use probability for fawns within the study area. We used fawn radiolocations (*N* = 2713; 2–56 locations/fawn) from birth to censor date, or 31 Aug to estimate resource use. We defined resource availability using the Geospatial Modelling Environment (Version 0.7.1.0; [49]) to generate an equal number of randomly distributed points using across a 100% minimum convex polygon of fawn radiolocations created using ArcGIS 10.0 [50]. We obtained raster-based vegetation data using 2006 National Landcover Data (30-m resolution; [51]) that we reclassified from 15 original landcovers to 8 (Table 2) and then converted to polygons using ArcGIS. We developed primary recreational vehicle trail data by traversing these trails with global positioning system units and converted these data to line shapefiles using ArcGIS. We obtained permanent water (i.e., river and lakeshore) and road data from Topologically Integrated Geographic Encoding and Referencing system files [52] and merged primary recreational vehicle trails with roads because roads and trails can affect deer behavior (e.g., predator risk avoidance; [5]). We used ArcGIS to spatially join radiolocations and random points to the vegetation data to identify the class and area of vegetation patch where each point was located. We used ArcGIS to estimate mean distance of each radiolocation found in a specific vegetation class to the edge of the nearest 3 patches of the same vegetation class (e.g., grassland to nearest 3 grassland patches). We used the nearest 3 patches to account for multiple patches which could be used for foraging and antipredator behaviors, such as hiding refugia [53], around a single radiolocation. We estimated distance to nearest road or permanent water source by conducting a spatial join between each radiolocation or random point and nearest road or permanent water source.

**Table 2:** Metrics used to assess resource use of neonatal white-tailed deer (≤ 14 weeks of age), southcentral Upper Peninsula of Michigan, USA, 2009–2011.

Metric	Definition
Lowland forest (%)	Forest with moist soil, periodically saturated with water and > 20% of total vegetation cover
Deciduous forest (%)	Forest with > 75% deciduous trees that are > 5 m tall and > 20% of total vegetation cover
Evergreen forest (%)	Forest with > 75% evergreen trees that are > 5 m tall and > 20% of total vegetation cover
Mixed forest (%)	Forest with a mix of deciduous and evergreen trees that individually comprise < 75% of total tree cover
Grass/shrub (%)	Vegetation > 80% graminoid or herbaceous, or trees or shrubs < 5 m tall
Pasture (%)	Grasses, legumes, or grass-legume mixtures for livestock grazing or production of seed or hay crop
Cropland (%)	Fields used for row crop (e.g., soybearn or corn) production, including orchards and land actively tilled
Wetland (%)	Soil is periodically saturated with or covered with water and is > 80% perennial herbaceous vegetation
Patch area (km^2^)	Geographic area of individual vegetation patch
Nearest patch (km^2^)	Mean distance of patch to edge of nearest 3 patches of same vegetation class
Distance to road (m)	Measure of the distance from a point of interest (e.g., deer radiolocation) to the edge of the nearest secondary or primary road, including intensively used motorized-vehicle trails
Distance to permanent water (m)	Measure of the distance from a point of interest (e.g., deer radiolocation) to the edge of the nearest permanent water source, including streams, rivers, and lake shores

We standardized all resource metrics to z-scores and centered scores to provide equal weight in multiple regression analyses [54]. We used variance inflation factor (VIF) analysis to assess multicollinearity among candidate resource metrics, with collinearity considered ≥ 7 [55]; no metrics were correlated (VIF = 1.04–2.62). We used package *lme4* [56] in R to assess binomial generalized linear mixed-effects models using a maximum likelihood estimator. We used radiolocations (1) and random points (0) as the binomial response variable and 8 vegetation classes, patch area, mean nearest patch, distance to nearest road, and distance to nearest permanent water as fixed effects with fawn and year as random effects on the intercept to account for variation among fawns and years [57]. We first evaluated a null model and models assessing individual parameters, and then additive models which included individually significant parameters (α = 0.05), including a global model of individually significant parameters. We used the receiver operating characteristic to estimate the area under the curve (AUC; [58]) to assess the predictive accuracy of models. We then ranked models by AUC estimates.

We used the Geospatial Modelling Environment (Version 0.7.1.0; [49]) to create a grid of contiguous square cells (2115 m^2^ /cell; mean telemetry error) across the area available to marked fawns. We then summarized the proportion of each vegetation class in each grid cell. We estimated the geometric centroid of each grid cell and calculated the patch area where the centroid was located, mean distance from centroid to nearest 3 similar vegetation patch classes, and distance from each centroid to nearest road or permanent water source. We used standardized coefficients from top ranked generalized linear mixed-models to spatially estimate a relative value of fawn resource suitability (*w*; [48]) for each grid cell:

$$w(x)=\exp({\beta_{0}+\beta }_{1}x_{1}+\beta_{2}x_{2}+\cdots +\beta_{k}x_{k}),$$ Eq 1

where *β*_k_ are the coefficients of the variables (*x*_*k*_). Summed coefficients could be a negative value or a value greater than 1, therefore we used a linear stretch [59] to limit fawn resource suitability (*w*) of each cell between 0 and 1:

$$\hat{w}=\left(\frac{w(x)-w_{min}}{w_{max}-w_{min}}\right),$$ Eq 2

where *w*_*min*_ and *w*_*max*_ represent the least and greatest resource use values, respectively. Each grid cell has a relatively greater likelihood of being used by fawns as its standardized values ($\hat{w}$) approaches 1. We joined resource suitability values to corresponding sampling grid cells shapefile and plotted the layer using ArcGIS.

### Predation risk

We used spatial models estimating likelihood of bobcat, black bear, coyote, and gray wolf predator resource selection in our study area (N. J. Svoboda, unpublished data) as surrogates of predation risk [5]. We derived resource selection functions from 23,135 to 101,874 global positioning system locations of 7 bobcats, 29 black bears, 21 coyotes, and 8 gray wolves from 25 May to 31 Aug 2009–2011 and used the functions to develop predator resource maps for these species. We clipped each predator resource selection map to the same grid configuration used for fawns and appended these values to matching fawn resource suitability grid cells to create species-specific predation risk maps. Similar to resource use, we used ArcGIS to clip fawn radiolocations from predation risk grid cells, which we summed to estimate integrated predation risk for each fawn radiolocation because each predator was attributed to a proportion of fawn mortalities in survival risk sets and may have influenced resource use of fawns.

### Winter severity

We estimated winter severity with a weather station that measured daily mean snow depth (cm), mean wind speed (kph), rainfall (cm), and minimum ambient temperature (C) near the center of the study area in a representative mixed coniferous and deciduous upland forest. We estimated a mean daily winter severity index by averaging the daily sum of snow depth, wind speed, and rainfall and subtracting that value from daily minimum temperature from 1 Jan to 31 Mar 2009–2011. We then summed daily winter severity values for the 3-month period each year and centered the data to 0, with greater severity with increasing positive numbers and less severity with increasing negative numbers. We developed this index because of minimal variation in snow depths and temperatures which were typically below levels used by other indexes (e.g.,[60]). We estimated the relative hazard of winter severity on fawn survival each year using package *survival* [61] in R 3.0 [62].

### Hiding cover

We estimated phenology of green vegetation growth using Normalized Difference Vegetation Index (250 m resolution; [63]) data as a surrogate for available hiding cover for fawns in open-canopy vegetation [64] during spring. We obtained 2009–2011 vegetation greenness values using the available 16 day composite data period closest to 1 Jun, when peak fawn parturition occurred during these years (Duquette, J.F., unpublished data). We used ArcGIS 10.0 [50] to clip vegetation data (19,883 pixels) to the study area and exported values to a spreadsheet.

### Survival analysis

We used Cox-proportional hazards in package *survival* [61] in R 3.0 [62] to estimate baseline seasonal fawn survival each year. Cox-proportional hazards models are semi-parametric regression models commonly used for survival data (e.g., [65]), which estimate proportional changes in the baseline survival hazard over time and relative differences in the hazard in relation to model covariates [66]. We modeled the survival of fawns using birth date of each fawn as the start time and date of censor, or 31 Aug as the stop time. We used the log-rank test using *α* = 0.05 to compare baseline survival estimates among years. We used mixed-effects models in package *coxme* [67] to assess if resource use, predator risk, body mass at birth, vegetation growth, and winter severity or additive models of these covariates best influenced fawn daily or seasonal survival and to account for variation in fawns among years. We assessed 12 daily (i.e., instantaneous) survival models using daily covariate values of radiolocations of fawns and 12 seasonal survival models using mean or median covariate values of radiolocations of fawns across the season from birth to censor, or 31 Aug. Plots of daily or seasonal fawn stop times and year showed clumped points associated with individual years, therefore we used individual fawn and year as random effects in all models. We estimated percent integrated deviance explained by subtracting the log-likelihood of an individual covariate model from the log-likelihood of the null model [68] and ranked models by deviance explained.

### Spatially-predictive mortality

We used the Geospatial Modelling Environment (Version 0.7.1.0; [49]) to create a grid of non-overlapping square cells (2115 m^2^/cell; mean telemetry error) across the landscape that was available to fawns. We spatially extrapolated survival coefficients from the non-ideal resource use and maternal effects model of daily survival by estimating survival rates to the end of each period (*S*[*te*]) as a function of resources or predation risk of each pixel according to:

$$S_{j}(t_{e}|x)={\left(S_{0,j}[t_{e}]\right)}^{exp(x\beta_{x})},$$ Eq 3

where (*S*_0,*j*_[*t*_*e*_]) is the baseline cumulative survival probability per year to 31 Aug, with different baseline estimates according to year, *j*, [61]. We then used a linear stretch (Eq 2; [59]) to constrain relative probability of fawn mortality between 0 and 1, with a greater likelihood of fawn mortality as standardized grid cell values approach 1. We appended resource suitability values to corresponding sampling grid cells shapefile and plotted the layer using ArcGIS.

## Results

### Fawn capture and monitoring

We obtained 2713 (median = 23, range = 2–89) radiolocations from 129 fawns. Coyotes were the primary (47%) predator, followed by bobcats (23%), black bears (8%), and wolves (8%), but the cause of remaining mortalities (14%) was unknown or other predators. In 2009, mean fawn birth body mass was 2.47 kg (SD = 0.78, *n* = 42), in 2010 was 4.16 kg (SD = 1.62, *n* = 35), and in 2011 was 4.11 kg (SD = 0.93, *n* = 47).

### Vegetation growth and winter severity

Mean vegetation growth in 2009 was 0.015 (SD = 0.929, *n* = 19882), in 2010 was 0.592 (SD = 0.796, *n* = 19882), and in 2011 was -0.607 (SD = 0.885, *n* = 19882). Greatest winter severity occurred in 2009 (455.9), followed by 2011 (242.5) and 2010 (-12.7). The proportional hazard of mortality increased with greater winter severity in 2009 (Hazard = 0.070) and 2011 (Hazard = 0.011), compared to 2010 (Hazard = 0.003).

### Resource use

We evaluated 17 models based on individual covariates of resource use (Table 3), with competing models including lowland forest, deciduous forest, coniferous forest, pasture, wetland, nearest patch distance, patch area, roads, and permanent water. We assessed 30 additive models of significant resources to estimate fawn resource suitability. The global model was the best model with an AUC of 0.82. The global model of fawn resource suitability suggested fawns used deciduous (*β* = 0.114, SE = 0.040, *P* = 0.004), greater distance between vegetation-specific patches (*β* = 0.765, SE = 0.043, *P* < 0.001), and locations closer to roads (*β* = -1.230, SE = 0.048, *P* < 0.001) and permanent water (*β* = 0.43, SE = 0.034, *P* < 0.001). Fawns avoided lowland forest (*β* = -0.213, SE = 0.050, *P* < 0.001), pastures (*β* = -0.119, SE = 0.040, *P* < 0.001), and wetlands (*β* = -0.087, SE = 0.042, *P* = 0.037). Coniferous forest (*β* = 0.035, SE = 0.034, *P* = 0.315) and vegetation patch area (*β* = -0.058, SE = 0.041, *P* = 0.157) were used in proportion to availability.

**Table 3:** Generalized linear mixed-effect models assessing second order resource use of neonatal white-tailed deer (≤ 14 weeks of age; *n* = 129) during the post-partum period (14 May–31Aug), southcentral Upper Peninsula of Michigan, USA, 2009–2011. Models used radiolocations (1; *n* = 2713) and random points (0) as the binomial response variable and individual resources were used as a fixed effect with individual fawn and year as random effects on the intercept. Model accuracy was estimated using the area under a receiver operating characteristic curve (AUC).

Parameters	Coefficient	Standard error	*z*-value	*P*-value	AUC
Distance to road (km)	-1.148	0.043	-27.026	< 0.001	0.77
Distance to water (km)	0.459	0.029	15.760	< 0.001	0.57
Nearest patch (km)	0.445	0.031	14.396	< 0.001	0.63
Lowland forest (%)	-0.283	0.027	-10.531	< 0.001	0.57
Deciduous forest (%)	0.141	0.027	5.223	< 0.001	0.53
Patch area (km)	-0.121	0.027	-4.516	< 0.001	0.57
Wetland (%)	-0.129	0.030	-4.311	< 0.001	0.51
Pasture (%)	0.075	0.027	2.801	0.005	0.51
Coniferous forest (%)	-0.068	0.027	-2.537	0.011	0.51
Grassland (%)	-0.025	0.027	-0.922	0.357	0.50
Mixed forest (%)	-0.017	0.027	-0.620	0.535	0.50
Cropland (%)	0.009	0.027	0.351	0.725	0.50

### Survival analysis

**Fig 1:**
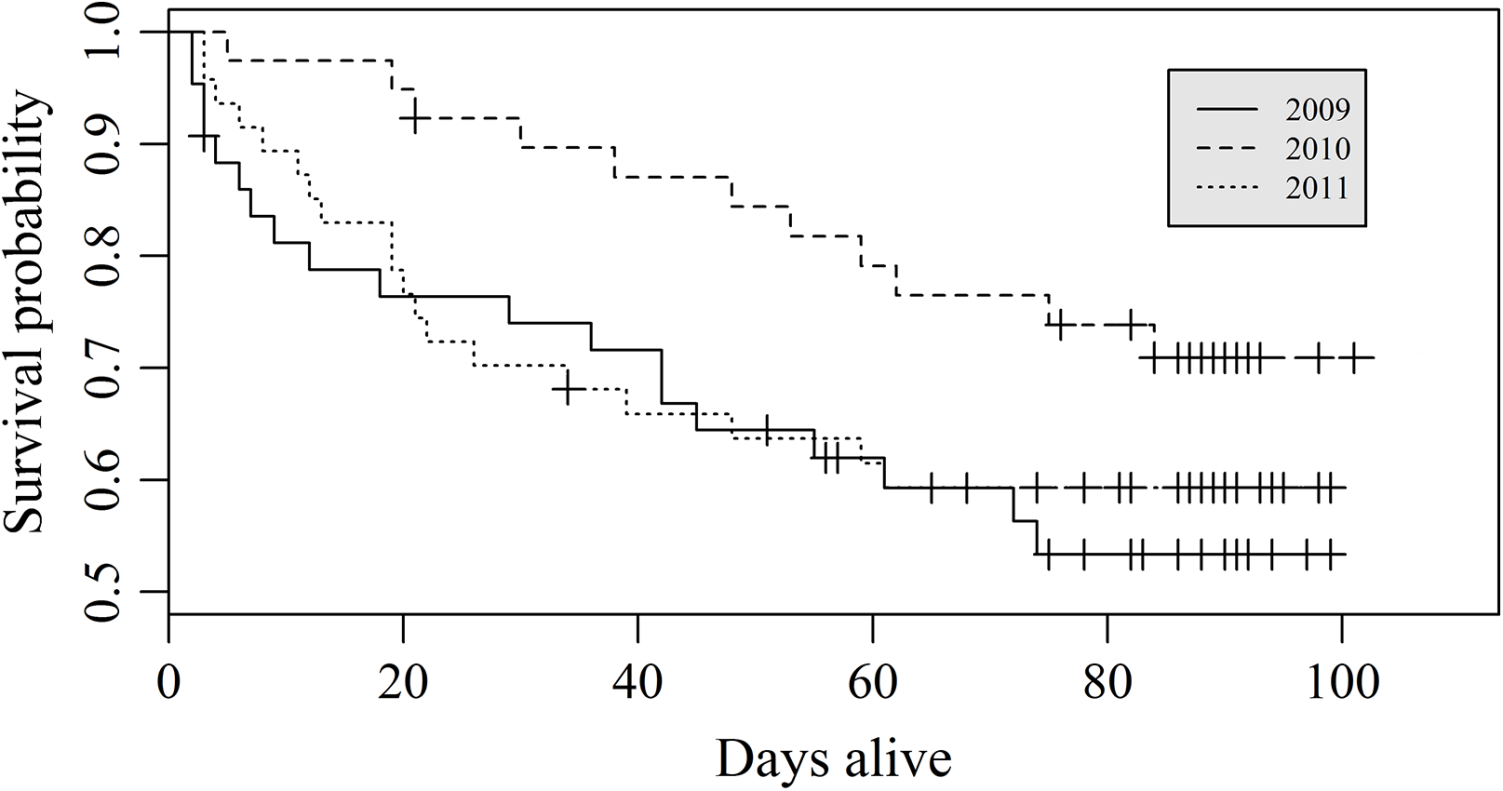
Kaplan–Meier estimates of neonate white-tailed deer fawn (≤ 14 weeks of age; *Odocoileus virginianus*; *n* = 129) survival from 14 May–31 August 2009–2011 in the southcentral Upper Peninsula of Michigan, USA.

Fawn survival was similar (*X* ^2^_2_ = 3.60, *P* = 0.168; Fig 1) among years. However, survival was less (*X* ^2^_1_ = 4.30, *P* = 0.038) in 2009 (0.52, SE = 0.08) than in 2010 (0.72, SE = 0.06), but similar (*X* ^2^_1_ = 0.20, *P* = 0.659) to 2011 (0.59, SE = 0.07). Survival was less (*X* ^2^_1_ = 4.10, *P* = 0.042) in 2011 than in 2010.

**Fig 2:**
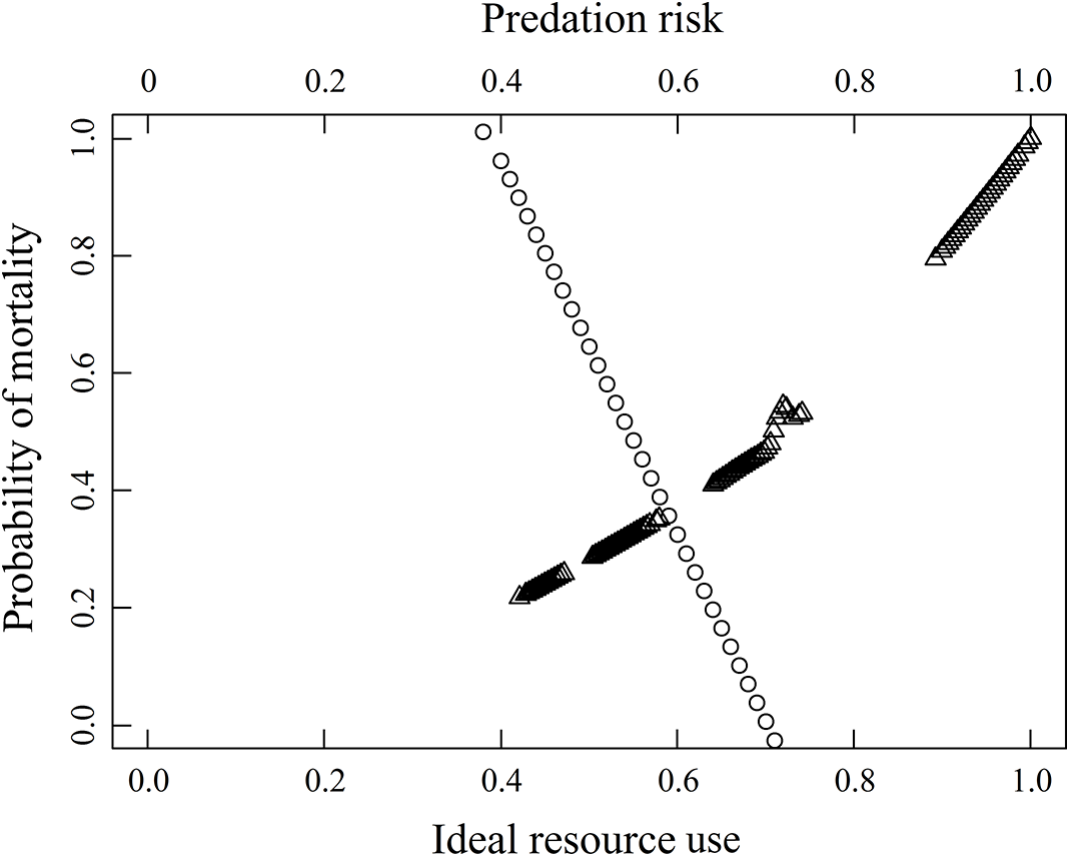
Cox-proportional hazards mixed-effects model assessing seasonally-averaged probability of mortality with ideal resource use (circles) and predation risk (triangles) of white-tailed deer fawns (≤ 14 weeks of age; *Odocoileus virginianus*; *n* = 129) captured as neonates during the maternal dependency period (14 May–31 August), southcentral Upper Peninsula of Michigan, USA, 2009–2011.

**Fig 3:**
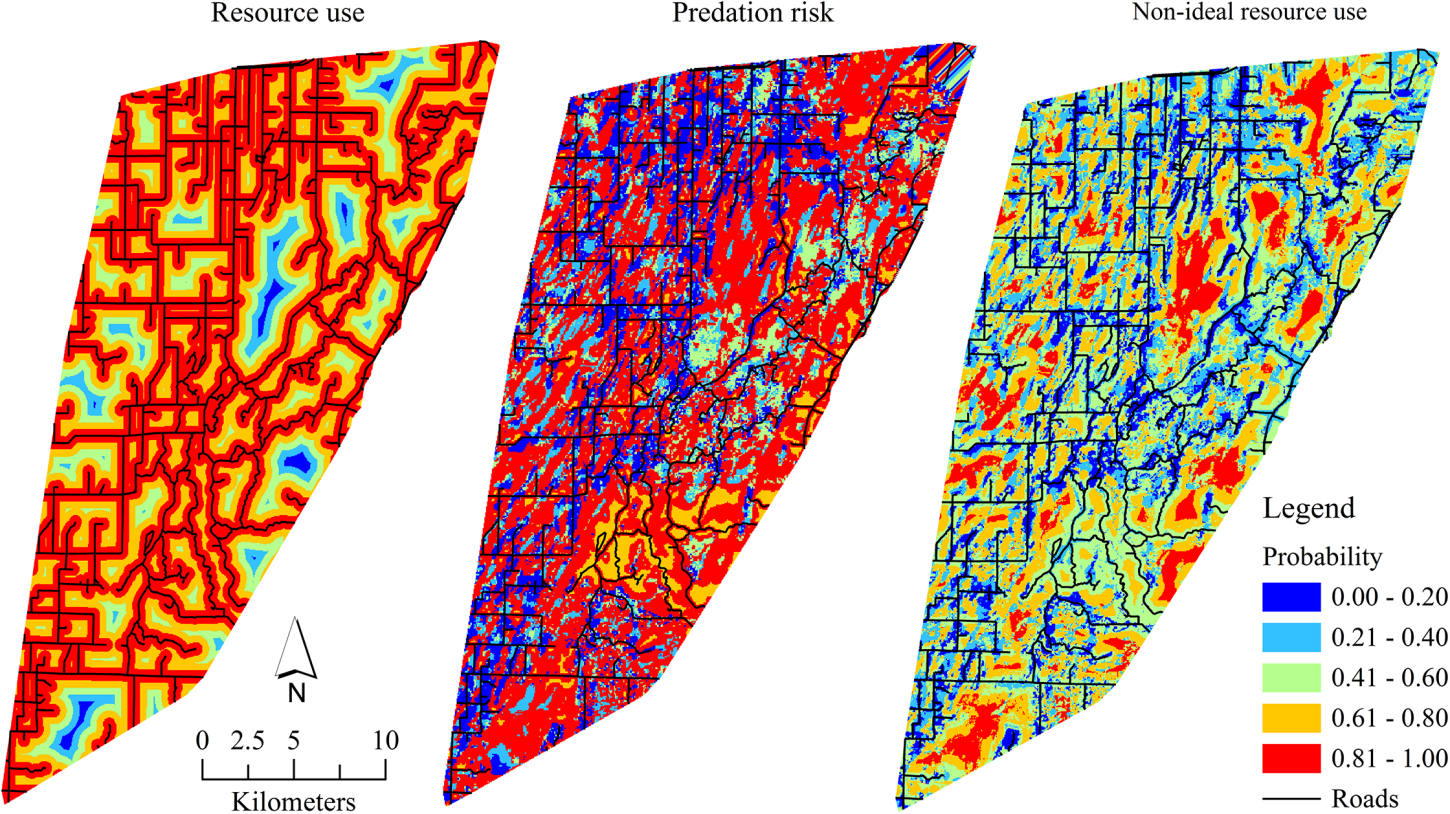
Spatially-predicted probability of resource use, integrated predation risk, and non-ideal resource use for white-tailed deer fawns (≤ 14 weeks old; *Odocoileus virginianus*; *n* = 129) captured as neonates during the maternal dependency period (25 May–31 August), Upper Peninsula of Michigan, USA, 2009–2011. Integrated predation risk was estimated from the summed probability of resource selection of bobcats (*Lynx rufus*), American black bears (*Ursus americanus*), coyotes (*Canis latrans*), and gray wolves (*C*. *lupus*).

We evaluated 17 models of daily or seasonal survival of fawns related to resource use, predation risk, maternal nutritional effects, winter weather, and hiding cover (Table 4). Daily survival of fawns was most influenced by non-ideal resource use and maternal nutritional effects that explained about 69% of the variation in daily survival, similar to the home range scale. However, maternal nutritional effects explained most (65%) of the variation in fawn daily survival. Parameter coefficients of the non-ideal resource use and maternal nutritional effects model at the landscape scale were of the same direction and similar magnitude as the home range scale (Table 5). A comparison of daily resource use and predation risk accounting for maternal nutritional effects showed likelihood of mortality increased linearly with resource use less than 59% or predation risk greater than 59% (Fig 2). Probability of fawn resource use extrapolated across the study area suggested resource use had a strong positive relationship with roads, but was negatively related to interior lowland forests (Fig 3). The predation risk model showed broad variation in risk across the study area, but increased risk appeared more spatially homogenous with greater lowland forest but less road density (i.e., interior forests). Non-ideal resource use of fawns extrapolated across the study area suggested that areas of decreased resource use suitability and increased predation risk had greater probability of mortality. The home range scale [13] similarly showed interior forests have increased probability of mortality, but to a lesser extent than at the landscape scale. Seasonal survival of fawns was most influenced by a weather-mediated ecological trap and maternal effects, but only explained 5.21% of the variation in seasonal survival and no model parameters were significant.

**Table 4:** Cox-proportional hazards mixed-effects models assessing the effects of resource use, predation risk, birth body mass, winter severity, and vegetation hiding cover on the daily or seasonal survival of white-tailed deer fawns (≤ 14 weeks of age; *n* = 129) during the post-partum period (14 May–31 Aug), Upper Peninsula of Michigan, USA, 2009–2011. Models included individual fawn and year as random effects on the intercept. Models presented with standardized parameter estimates, standard errors (SE), coefficient probability values, degrees of freedom (*df*), and estimated hazard ratio parameter probability values, and percent integrated deviance explained indicating the reduction in the log-likelihood from the null model. Percent deviance explained was used to rank models. Model fit was assessed using a Chi-square test of log-likelihood of a given model (Log-likelihood *X* ^2^) compared to the null model.

Model	Coefficient	SE	*P*-value	Hazard ratio	Deviance explained (%)	Log-likelihood *X* ^2^	Model *P*-value
Daily survival (*n* = 2695)							
**Non-ideal use + maternal effects**					69.34	138.67	< 0.001
Resource use	-0.705	0.256	0.006	0.494			
Predation risk	-0.227	0.225	0.310	0.797			
Birth body mass	-2.74	0.065	< 0.001	0.065			
Winter severity	0.139	0.484	0.770	1.149			
Body mass * Winter severity	-0.777	0.326	0.017	0.46			
**Ecological trap + maternal effects**					69.29	138.58	< 0.001
Resource use	-0.699	0.27	0.010	0.497			
Predation risk	-0.228	0.226	0.310	0.796			
Resource use * predation risk	-0.014	0.212	0.950	0.986			
Birth body mass	-2.734	0.531	< 0.001	0.065			
Winter severity	0.137	0.482	0.780	1.146			
Body mass * Winter severity	-0.775	0.325	0.017	0.461			
**Weather-mediated ecological trap + maternal effects**					68.69	137.38	< 0.001
Resource use	-0.691	0.269	0.010	0.501			
Predation risk	-0.224	0.225	0.320	0.799			
Resource use * predation risk	-0.010	0.213	0.960	0.990			
Birth body mass	-2.706	0.523	< 0.001	0.067			
Winter severity	0.136	0.485	0.780	1.146			
Body mass * Winter severity	-0.782	0.322	0.015	0.458			
Predation risk * Winter severity	-0.017	0.192	0.930	0.983			
**Resource use + maternal effects**					68.24	135.70	< 0.001
Resource use	-0.558	0.211	0.008	0.572			
Birth body mass	-2.723	0.529	< 0.001	0.066			
Winter severity	0.112	0.489	0.820	1.118			
Body mass * Winter severity	-0.791	0.327	0.015	0.453			
**Maternal effects**					64.85	129.70	< 0.001
Birth body mass	-2.738	0.548	< 0.001	0.065			
Winter severity	0.220	0.497	0.660	1.246			
Body mass * Winter severity	-0.817	0.334	0.015	0.442			
**Predation risk + maternal effects**					64.68	128.86	< 0.001
Predation risk	0.111	0.193	0.570	1.117			
Birth body mass	-2.694	0.545	< 0.001	0.068			
Winter severity	0.125	0.51	0.810	1.133			
Body mass * Winter severity	-0.829	0.339	0.014	0.436			
**Birth body mass**	-2.627	0.489	< 0.001	0.072	59.96	119.92	< 0.001
**Ecological trap + hiding cover**					47.19	94.38	< 0.001
Resource use	-0.645	0.262	0.014	0.525			
Predation risk	-0.192	0.217	0.380	0.826			
Resource use * predation risk	-0.028	0.211	0.900	0.973			
Hiding cover	-0.327	0.487	0.500	0.721			
**Ecological trap**					46.36	92.72	< 0.001
Resource use	-0.66	0.259	0.011	0.517			
Predation risk	-0.2001	0.217	0.350	0.818			
Resource use * predation risk	-0.022	0.211	0.920	0.21			
**Non-ideal use**					46.30	92.60	< 0.001
Resource use	-0.668	0.244	0.006	0.513			
Predation risk	-0.201	0.215	0.350	0.818			
**Non-ideal use + hiding cover**					46.25	92.51	< 0.001
Resource use	-0.650	0.245	0.008	0.522			
Predation risk	-0.173	0.215	0.420	0.841			
Hiding cover	-0.489	0.498	0.330	0.613			
**Resource use**	-0.546	0.203	0.007	0.579	45.66	91.32	< 0.001
**Winter severity**	1.158	0.415	0.005	3.183	44.32	88.64	< 0.001
**Weather-mediated predation**					44.13	88.26	< 0.001
Predation risk	0.106	0.187	0.570	1.111			
Winter severity	1.152	3.164	0.009	3.164			
Predation risk * Winter severity	-0.025	0.196	0.900	0.975			
**Hiding cover**	-0.472	0.508	0.350	0.624	42.23	84.46	< 0.001
**Predation risk**	0.129	0.183	0.480	1.138	40.43	80.86	< 0.001
**Null**	-	-	-	-	38.76	77.51	< 0.001
Seasonal survival (*n* = 124)							
**Weather-mediated ecological trap + maternal effects**					5.21	9.90	0.194
Resource use	-0.185	0.268	0.490	0.831			
Predation risk	-0.013	0.250	0.960	0.987			
Resource use * predation risk	0.392	0.251	0.120	1.480			
Birth body mass	-0.004	0.203	0.990	0.996			
Winter severity	0.323	0.249	0.190	1.382			
Body mass * Winter severity	-0.130	0.189	0.490	0.878			
Predation risk * Winter severity	0.423	0.236	0.074	1.526			
**Ecological trap + maternal effects**					3.68	7.35	0.499
Resource use	-0.180	0.264	0.490	0.835			
Predation risk	0.075	0.242	0.760	1.078			
Resource use * predation risk	0.287	0.215	0.180	1.333			
Birth body mass	0.031	0.196	0.870	1.032			
Winter severity	0.283	0.212	0.180	1.327			
Body mass * Winter severity	-0.183	0.168	0.280	0.833			
**Weather-mediated predation**					3.36	6.19	0.103
Predation risk	0.020	0.196	0.920	1.014			
Winter severity	0.316	0.178	0.075	1.343			
Predation risk * Winter severity	0.335	0.195	0.085	1.437			
**Ecological trap + hiding cover**					3.05	6.11	0.412
Resource use	0.029	0.260	0.910	1.029			
Predation risk	0.228	0.234	0.330	1.257			
Resource use * predation risk	0.183	0.183	0.320	1.200			
Hiding cover	-0.465	0.277	0.093	0.628			
**Non-ideal use + maternal effects**					2.57	5.13	0.644
Resource use	0.065	0.189	0.730	1.067			
Predation risk	0.165	0.234	0.480	1.179			
Birth body mass	0.045	0.197	0.820	1.045			
Winter severity	0.284	0.211	0.180	1.329			
Body mass * Winter severity	-0.170	0.167	0.310	0.844			
**Predation risk + maternal effects**					2.51	5.02	0.542
Predation risk	0.118	0.191	0.540	1.125			
Birth body mass	0.035	0.194	0.860	1.036			
Winter severity	0.278	0.210	0.190	1.320			
Body mass * Winter severity	-0.169	0.166	0.310	0.845			
**Non-ideal use + hiding cover**					2.49	4.97	0.419
Resource use	0.205	0.193	0.290	1.227			
Predation risk	0.296	0.226	0.190	1.345			
Hiding cover	-0.468	0.279	0.094	0.626			
**Resource use + maternal effects**					2.32	4.63	0.592
Resource use	-0.012	0.151	0.930	0.987			
Birth body mass	0.028	0.193	0.880	1.028			
Winter severity	0.300	0.206	0.150	1.350			
Body mass * Winter severity	-0.158	0.164	0.330	0.854			
**Maternal effects**					2.31	4.62	0.464
Birth body mass	0.029	0.193	0.880	1.030			
Winter severity	0.304	0.203	0.140	1.355			
Body mass * Winter severity	-0.158	0.164	0.340	0.854			
**Winter severity**	0.294	0.156	0.059	1.341	1.81	3.61	0.307
**Hiding cover**	-0.390	0.248	0.120	0.677	1.52	3.05	0.385
**Ecological trap**					1.21	2.42	0.789
Resource use	-0.089	0.242	0.720	0.915			
Predation risk	0.135	0.226	0.550	1.145			
Resource use * predation risk	0.180	0.182	0.320	1.198			
**Non-ideal use**					0.65	1.3	0.861
Resource use	0.076	0.175	0.670	1.079			
Predation risk	0.204	0.218	0.350	1.227			
**Predation risk**	0.154	0.180	0.390	1.166	0.56	1.11	0.775
**Birth body mass**	-0.067	0.169	0.690	0.936	0.33	0.67	0.881
**Null**	-	-	-	-	0.24	0.47	0.789
**Resource use**	-0.008	0.137	0.950	0.992	0.22	0.44	0.932

**Table 5:** Comparison of the best cox-proportional hazards mixed-effects models assessing the effects of resource use, predation risk, birth body mass, winter severity, and vegetation hiding cover on the daily survival of white-tailed deer fawns (≤ 14 weeks of age; *n* = 129) at the home range and landscape scales during the post-partum period (14 May–31 Aug), Upper Peninsula of Michigan, USA, 2009–2011. Models included individual fawn and year as random effects on the intercept. Models presented with standardized parameter estimates, standard errors (SE), probability values, and estimated hazard ratio parameter probability values, and percent integrated deviance explained indicating the reduction in the log-likelihood from the null model. The home range model was available from [13].

Model	Estimate	SE	Coefficient *P*-value	Hazard ratio	Deviance explained (%)
Home range scale					
**Non-ideal use + maternal effects**					70.78
Resource use	-0.561	0.194	< 0.001	0.571	
Predation risk	0.165	0.211	0.430	1.179	
Birth body mass	-2.784	0.539	< 0.001	0.062	
Winter severity	0.146	0.501	0.770	1.157	
Body mass * Winter severity	-0.811	0.330	0.014	0.444	
Landscape scale					
**Non-ideal use + maternal effects**					69.34
Resource use	-0.705	0.256	0.006	0.494	
Predation risk	-0.227	0.225	0.310	0.797	
Birth body mass	-2.740	0.065	< 0.001	0.065	
Winter severity	0.139	0.484	0.770	1.149	
Body mass * Winter severity	-0.777	0.326	0.017	0.460	

## Discussion

Fawn survival was most influenced by dam nutritional condition and winter weather relative to daily behavioral trade-offs in resource selection and multi-predator risk across the landscape. Although maternal nutrition and non-ideal resource use had additive effects on fawn survival, maternal nutritional effects on birth body mass of fawns explained most (65%) of the variation in survival. These results were similarly supported by concomitant research [13] at the home range scale, where maternal nutrition explained 64% of the variation in daily fawn survival. Therefore, it appears that nutritional condition was the primary factor influencing fawn survival at landscape (this study) and home range scales, with non-ideal resource use potentially limiting survival at finer spatial scales. Similar nutritional [16] or scale-dependent trade-offs in resource selection and predator avoidance [7, 18, 31] have been reported with white-tailed deer and other ungulates. Mutual support of our results suggests adult female reproductive success was influenced by variation in similar factors occurring at multiple spatial scales, not supporting the prediction of broad-scale limitation by [1].

Similar support of resource use, multi-predator risk, and nutritional effects limiting fawn survival not only occurred across spatial scales, but also temporal scales [13]. Nutritional effects, and to a lesser extent non-ideal resource use, strongly affected the daily mortality rate for fawns compared to these effects averaged over the post-partum period across the landscape (this study). Daily variation in these effects likely had greater influence than averaged effects over the period on fawn survival than did seasonal variation because mean age of fawns at mortality was 64 days, while the period was 109 days. Additionally, while seasonally averaged survival was best explained by a weather-mediated ecological trap and nutritional effects, this model likely poorly explained survival because the effects of weather on ungulate survival are not typically a proximate cause of mortality, but rather the ultimate cause of mortality through influences on nutritional condition and predation risk [12]. The importance of dam daily trade-offs in resource selection and predator avoidance are highlighted by the relatively minor variation in resource use and predator avoidance behaviors sharply changing the likelihood of fawn survival. Sensitivity of fawn survival to daily variation in dam maternal behaviors suggests that surviving fawns were born to dams which were more successful at balancing daily variation in resource selection and multi-predator risk during the first 2 months post-partum. Nonetheless, dam selection and avoidance behaviors were secondary to the effects of winter weather limiting their nutritional condition.

Maternal nutritional carry-over effects are common in ungulates [23, 69] and can predispose neonates with poorer nutritional condition to greater mortality risk, particularly predation [16, 22]. Maternal nutritional effects during our study were related to yearly variation in winter weather preceding parturition, which decreased birth body mass and survival of fawns following more severe winters [16, 22]. Decreased body mass would have made fawns less able to physically evade predators [47] and increased the mortality risk for fawns using areas with poor hiding cover and greater predation risk. As most (75%) mortalities were attributed to predation within a mean of 31 days (range = 2–84) of birth, the physical ability of fawns to move and avoid predators was presumably limited. Additionally, increased nutritional demands following more severe winters could have exacerbated the influence of non-ideal resource use on fawn survival, whereas dams selected vegetation which placated their nutritional needs at the expense of exposing fawns to greater predation risk [11]. While winters during our study were relatively mild compared to studies of white-tailed deer in similar latitudes (e.g., [70]), wildlife managers should recognize that these winter conditions were enough to influence population growth of deer [37].

The cascading influence of winter weather on daily maternal nutritional effects was emphasized by the similar inverse relationship at the home range [13] and landscape (this study) scales. The strong relationship between winter weather and maternal nutrition effects was exemplified by least survival and greatest winter severity in 2009, and greatest survival and least winter severity in 2010. However, there appeared to be a threshold of winter severity which increased the fawn mortality hazard nearly 7-fold between the 2011 and 2009 estimates. We could not identify the threshold value at which mortality increased, but fawn mortality appeared to increase linearly with winter severity values greater than in 2011. Although we predicted winter severity would also indirectly affect fawn survival by influencing the timing of vegetation growth needed for hiding cover during spring [14, 64, 71, 72], minimal variation in vegetation growth around peak parturition (1 June) likely dampened its influence on survival. Nonetheless, the pattern in vegetation growth across years was inversely related with fawn predation rates which decreased 29% from 2009 to 2010, but increased 67% from 2010 to 2011 when preceding winter severity was greater. Similar body mass at birth between 2010 and 2011 also suggested hiding cover likely had a partial role in predation rates in these years, beyond the influence of fawn body mass.

Although non-ideal resource use explained less variation than nutritional condition in survival of fawns, overall support for this model across spatial scales [13] suggested that dams placed fawns [71] in poor resources (e.g., meager hiding cover) where predation was additive beyond those related to the resources alone [7, 43]. However, resource use explained most of the variation in non-ideal resource use, as predation risk was not significant in any models and did not mediate resource use, as would be assumed in an ecological trap [42]. Hence, the ability of dams to perceive cues in resource quality on a daily basis was more influential than predation risk to survival of fawns [7, 43]. Coefficients of non-ideal resource use suggested dams avoided raising fawns in wetlands and coniferous forest possibly due to poor thermoregulatory vegetation and also avoided pastures likely due to less visual concealment from predators during spring [27]. Dams instead raised fawns in isolated deciduous forest patches near roads and permanent water (i.e., rivers and streams), which could have provided adequate vegetation to meet their nutritional needs [11, 70, 73] while providing isolated refugia cover to improve detection and avoidance of predators [14, 74]. These resources also likely allowed dams to avoid overlapping resources with their primary predator, wolves [75, 76]. Core wolf territories in our study area were located in interior lowland forests [76; N. J. Svoboda, unpublished data], which dams possibly perceived as areas of greater direct mortality risk [77, 78]. However, by dams selecting parturition areas in patches of refuge cover from wolves, fawns were likely exposed to greater predation risk from alternative predators, particularly coyotes. Similar resource use occurred across years and within fawn home ranges [13], suggesting dams were selecting areas with adequate forage and avoiding wolves at the expense of losing fawns to alternative predators in some years [5]. This maternal behavior strategy was likely important to improving the life-time reproductive success of dams [17, 21], particularly as females up to 15.5 years old were pregnant [79] and could have produced multiple litters of fawns.

While predation was the leading cause of fawn mortality, variation in integrated predation risk did not appear to influence fawn resource use at the home range [13] or landscape (this study) scales. Nonetheless, species-specific predation rates suggested variation in predation risk can directly [80] or indirectly [11, 81, 82] influence neonatal ungulate survival through maternal behavioral trade-offs between resource selection and predation risk. We recognize our understanding of the relationship between fawn survival and predation risk was limited because our predation risk data was based on probability of fawns encountering predators, rather than probability of fawns actually being killed [5]. Also, too few mortalities of radiomarked dams occurred across years to compare their survival to resource selection and predator avoidance strategies, which limited our interpretation of these behaviors related to reproductive success [21]. Nonetheless, survival (70%) of radiomarked adult females across years was greater than fawns [37] and supported our interpretations that dams used resource selection and predator avoidance strategies to maximize their nutritional condition and reduce their own mortality risk. As the order of factors limiting adult female reproductive success was nutritional condition, then resource use, and then multi-predator risk at the landscape (this study) and home range [13] scales, we suggest that wildlife managers should consider increasing year-round forage quality and heterogeneity [83] at both spatial scales. This habitat management regimen could help to increase fawn survival during the post-partum period by increasing fawn nutritional condition and reduce predation risk by increasing the abundance and dispersion of hiding cover.

## Supporting Information

S1 Dataset Data used to assess the seasonal survival of white-tailed deer fawns (≤ 14 weeks of age; *Odocoileus virginianus*; *n* = 129) during the post-partum period (14 May–31 Aug), Upper Peninsula of Michigan, USA, 2009–2011. (CSV)

S2 Dataset Data used to assess the daily survival of white-tailed deer fawns (≤ 14 weeks of age; *Odocoileus virginianus*; *n* = 129) during the post-partum period (14 May–31 Aug), Upper Peninsula of Michigan, USA, 2009–2011. (CSV)

S1 Fig Location (black polygon) of adult female white-tailed deer (*Odocoileus virginianus*) reproductive success study, Upper Peninsula of Michigan, USA, 2009–2011. (TIF)
