# Supplementary figures and images for: Scale Dependence of Female Ungulate Reproductive Success in Relation to Nutritional Condition, Resource Selection and Multi-Predator Avoidance
Source: PLoS One. 2015 Oct 16;10(10):e0140433. doi: 10.1371/journal.pone.0140433 (PMC4608707; doi:10.1371/journal.pone.0140433)

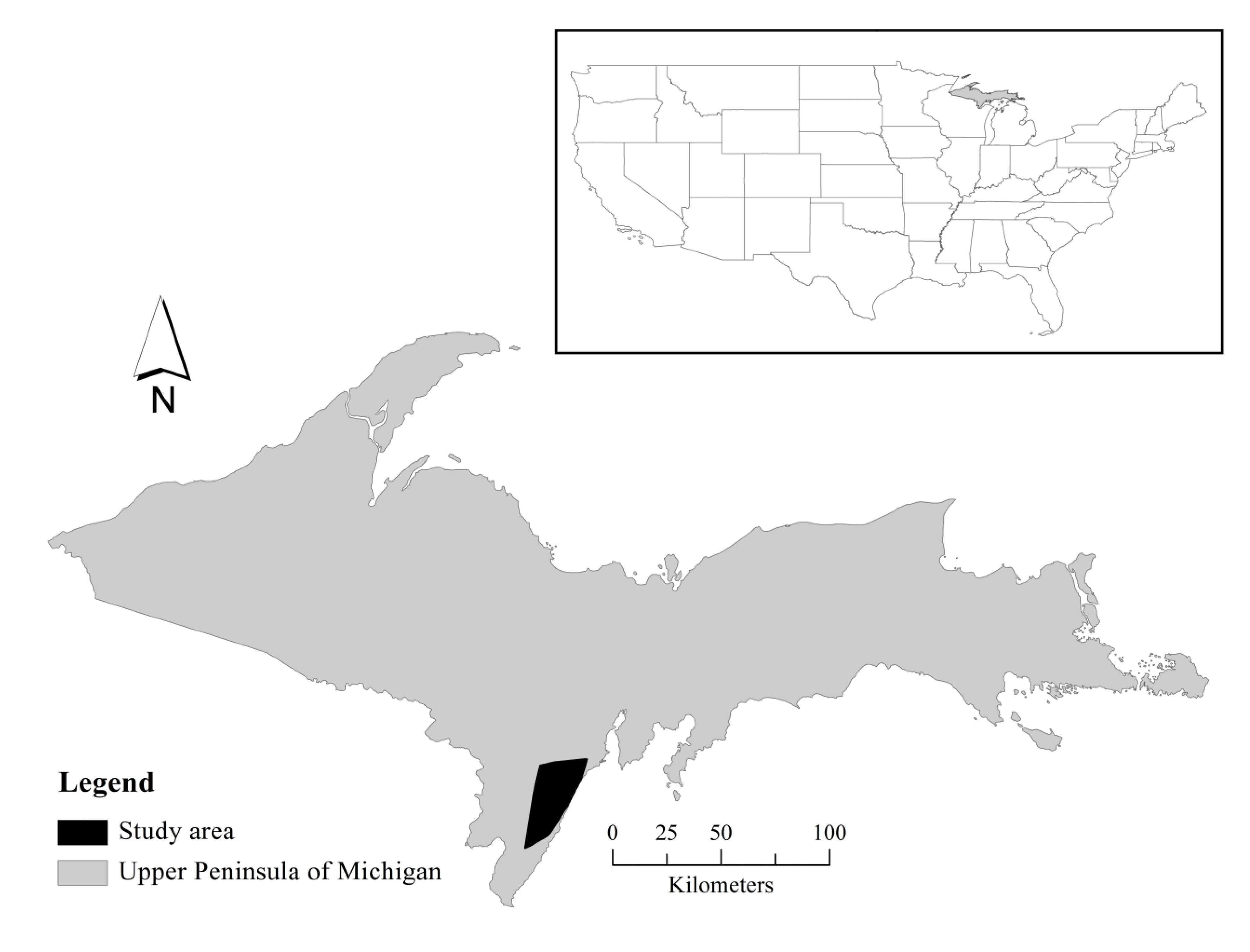

Supplement: S1 Fig — (TIF) [file pone.0140433.s003.tif]
